# Creating Personalized Recommendations in a Smart Community by Performing User Trajectory Analysis through Social Internet of Things Deployment

**DOI:** 10.3390/s20072098

**Published:** 2020-04-08

**Authors:** Guang Xing Lye, Wai Khuen Cheng, Teik Boon Tan, Chen Wei Hung, Yen-Lin Chen

**Affiliations:** 1 Faculty of Information and Communication Technology, Universiti Tunku Abdul Rahman, 31900 Kampar, Perak, Malaysia; simple@1utar.my (G.X.L.); chengwk@utar.edu.my (W.K.C.); tantb@utar.edu.my (T.B.T.); hungcw@utar.edu.my (C.W.H.); 2 Department of Computer Science and Information Engineering, National Taipei University of Technology, 1, Sec. 3, Chung-hsiao E. Rd., Taipei 10608, Taiwan

**Keywords:** personalized recommendation, user trajectory analysis, Social Internet of Things (SIoT), service discovery, recommender engine, smart community

## Abstract

Despite advancements in the Internet of Things (IoT) and social networks, developing an intelligent service discovery and composition framework in the Social IoT (SIoT) domain remains a challenge. In the IoT, a large number of things are connected together according to the different objectives of their owners. Due to this extensive connection of heterogeneous objects, generating a suitable recommendation for users becomes very difficult. The complexity of this problem exponentially increases when additional issues, such as user preferences, autonomous settings, and a chaotic IoT environment, must be considered. For the aforementioned reasons, this paper presents an SIoT architecture with a personalized recommendation framework to enhance service discovery and composition. The novel contribution of this study is the development of a unique personalized recommender engine that is based on the knowledge–desire–intention model and is suitable for service discovery in a smart community. Our algorithm provides service recommendations with high satisfaction by analyzing data concerning users’ beliefs and surroundings. Moreover, the algorithm eliminates the prevalent cold start problem in the early stage of recommendation generation. Several experiments and benchmarking on different datasets are conducted to investigate the performance of the proposed personalized recommender engine. The experimental precision and recall results indicate that the proposed approach can achieve up to an approximately 28% higher F-score than conventional approaches. In general, the proposed hybrid approach outperforms other methods.

## 1. Introduction

The Social Internet of Things (SIoT) is an emerging paradigm of the Internet of Things (IoT) in which heterogeneous IoT devices can communicate with each other, collaborate on behalf of their owners, establish relationships based on common interests, and autonomously perform service trading. SIoT is expected to enhance the features of existing distributed systems, such as service discovery and composition [1,2,3], information management [4,5,6,7], and service trustworthiness management [8,9,10]. Although SIoT has begun to be adopted in some domains, such as smart vehicles [11,12,13,14], smart homes [15], smart factories [16], and integrated transportation [17], current SIoT systems encounter numerous challenges that affect their usability and reliability in existing SIoT domains [18,19]. 

In general, IoT applications are developed to solve specific problems and usually do not share and use data from other IoT services to generate recommendations. Thus, the coordination among IoT services is inefficient because efforts to obtain similar datasets overlap [20]. SIoT systems can improve coordination among IoT services because these systems comprise an object profile based on the IoT data and accessibility of each IoT device or component. SIoT networks enable objects to establish social relationships autonomously and thereby gain object popularity through coordination. These objects perform data exchange by joining different SIoT networks. SIoT networks can provide recommendation services to different IoT applications by referring to the data accessibility in each object profile. The content of the object profile gradually improves over time according to its owner’s experiences with and feedback on each previous recommendation. This demonstrates the importance of making credible and quality recommendations as a means of acquiring the object equivalent of social capital and attaining object popularity.

**Figure 1:**
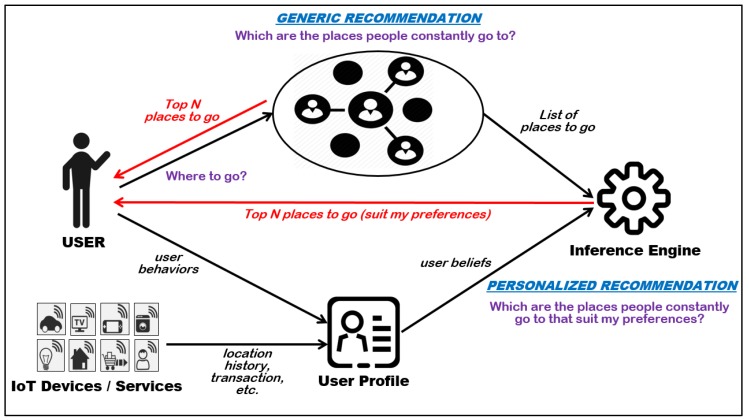
Differences between generic and personalized recommendations.

Personalization and recommendation are two key prerequisites in SIoT systems that enable delivery of a promising service [21]. Both prerequisites are essential to producing a high satisfaction level for SIoT solution that matches the preferences of the user. IoT applications in the community should establish trust to ensure reliable interactions between relevant stakeholders to reduce exposure to malicious entities. A crucial problem in personalized recommendations is the generation of alternative solutions when the service provider fails to provide the requested service. Furthermore, the alternative solution must be calibrated to fulfill the preferences of the user. The aforementioned problem has a broad scope and poses greater challenges to existing recommender systems. The difference between generic and personalized recommendation is shown in Figure 1, as personalized recommendation requires input from a user profile to infer relevant output to a user. A user profile contains relevant personal data (e.g., user behavior, location history, and transaction) collected from different sources (e.g., user activities, IoT devices, and service interactions), and allows recommendation by referring to user preferences.

A recommender system is mainly based on information discovery and information filtering. According to Bobadilla et al. [22], a recommendation is influenced by the data collection method (including the data preprocessing and ranking methods), data filtering algorithm (e.g., content-based, collaborative, and hybrid algorithms), selected data model (e.g., memory-based and model-based methods), techniques employed for reasoning (e.g., probabilistic approaches and neural networks), data sparsity, and system performance management. According to our previous study [23], location-based smart information systems can use the mobile trajectories of users to recommend several points of interest according to user preferences and conditions. The trajectory data can be obtained from relevant sensors embedded in mobile phones, wearable gadgets, and smart environment (e.g., buildings, and check-in points). Many existing solutions detailed in the literature focus on modifying the service layer. These solutions involve providing recommendations to users based on static information, such as preloaded service details (e.g., location and type) in an area and the current user position. Often, limited choices are generated that do not comport with actual human needs. 

The major contribution of this article is a personalized recommendation system suitable for service discovery in a smart community, specifically SIoT networks. In particular, the novelty of this study lies in the following aspects:

(a) a trajectory analysis framework that applies user location histories, specifically the trajectories of users with similar behavior and movement patterns,
(b) the adoption of the knowledge–desire–intention (KDI) model [23] to collect user data explicitly (e.g., ratings for items) and implicitly (e.g., location history and number of orders) from profile users, and
(c) a hybrid reasoning approach to leverage the available trajectory-based and contextualized data in performing personalized recommendations.

We adopt the link analysis (LA) method proposed by Zheng et al. [24,25] to capture the location correlation to achieve more effective and accurate item-based collaborative filtering (CF) [26], which can generate both generic and personalized recommendations. However, our framework is different from that of Zheng et al. in three aspects. First, we adopt KDI hierarchical belief modeling [27] for user profile. The Slope One algorithm [24] is applied with a simple linear regression model to solve the recommendation problem. Second, we use a user feedback mechanism for fine-tuning items’ weight vectors after each session of recommendation generation to avoid the issue of local optimization. Third, the proposed framework is based on domain-independent user trajectory analysis, which is suitable for all types of IoT applications. Such a framework is appropriate for SIoT environments with various domains of intelligent systems that can interact closely. 

Besides, we examine the proposed personalized recommendation framework in two stages. In a previous study, we investigate the performance of the recommender engine in terms of its ability to handle a smart campus dataset from UniCAT [23]. We also conduct a study previously in which we experimentally investigate the characteristics of various filtering algorithms for recommending a place to visit. In the experiment, the trajectory records of 100 active users over 1 year are used for evaluation. The UniCAT dataset contains many new student profiles and only a limited number of user trajectory records, representing a cold start scenario. Under the same settings, our hybrid approach outperformed the baseline and CF methods (the proposed approach had higher precision and recall). This result indicates that the accuracy of the CF method can be improved using a more sophisticated knowledge base to support the personalization process. An increase in accuracy allows an improvement in the satisfaction with the overall result. In the second stage, we enlarge the scale of the recommender engine through several experiments and benchmarking processes to support different datasets. The four selected datasets, namely GeoLife [28], Weeplaces [29], Brightkite [30], and Gowalla [29], suitably represented the application of intelligent services in a smart community. The experimental precision and recall results indicated that the proposed hybrid technique can achieve up to an approximately 28% higher F-score than conventional approaches can. In general, the proposed personalized recommendation method outperforms other methods.

The rest of this paper is organized as follows: Section 2 introduces the background of the study that involves SIoT architecture and a use case scenario, and various recommendation methodologies. Section 3 illustrates the overall implementation and challenges of personalized user trajectory analysis in a smart community. Section 4 describes the proposed personalized recommendation framework for smart communities, including its relevant components. Section 5 describes the implementation of the proposed SIoT system as well as comparison and measurement criteria. Section 6 presents the experimental results and a comparison of the proposed method with several benchmarking approaches. Section 7 outlines the conclusions and future research directions. 

## 2. Background

### 2.1. SIoT

Lately, developing the paradigm of an ecosystem that can enable users and smart objects to interact within a social framework has received considerable attention from the academic and industry sectors [6,9,10,31,32]. In general, a social relationship exists among smart objects, the digital space, and users. User–object and object–object social relationships have evolved into SIoT, which imitates traditional social networking operations and features to establish relationships between IoT applications [21]. For instance, through data sharing and web technologies in SIoT platform, service providers in a smart community can offer various user-friendly IoT services (this process results in the development of strong user–object and object–object relationships). These smart objects connect through different SIoT network structures that allow them to perform autonomous and proactive interactions with other users and objects. Additional data or information shared by other IoT applications with an IoT service can further enhance and enrich the recommendation service. This article proposes a new framework to integrate the data from different IoT services. 

**Figure 2:**
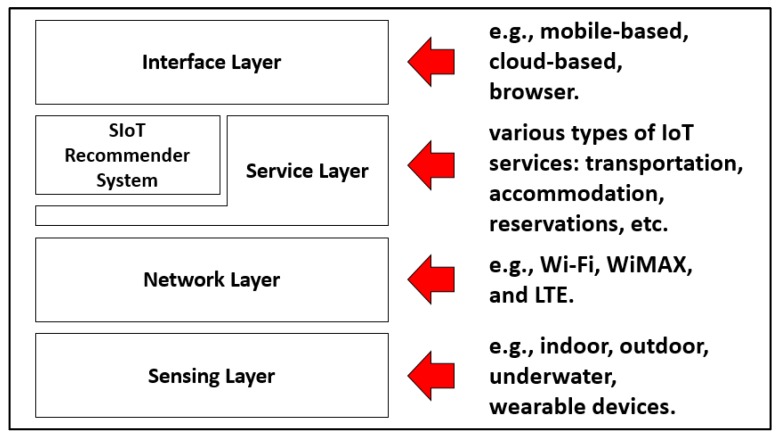
Proposed SIoT architecture with the recommender module.

State-of-the-art SIoT research has reviewed different SIoT platforms and architectures, service discovery and composition, relationship management, network navigability, and trustworthiness management in SIoT environments [19,21,33]. For service discovery, the interest similarity among users and the frequency of interaction between smart devices are considered to determine relevant social objects. Details such as demographic information, friendly relationships, and user interests are considered in the process of generating service recommendations [34]. Some studies have integrated various approaches to provide service recommendations [35]. However, these methods are still very domain-specific and require extensive study for further SIoT deployment. In this study, we select SIoT domain to demonstrate the proposed personalized recommendation framework because the framework has a suitable object profile construction capability and service recommendation function. These two features are the core elements of an SIoT platform. The proposed SIoT architecture is displayed in Figure 2.

In general, the user and things layers are clearly defined in an SIoT architecture. SIoT platforms enable things to establish social networks, and users can impose different policies to control the accessibility of interconnected autonomous entities. A new type of service delivery is the outcome of automated service composition between the things. We adopt SIoT architecture illustrated in Figure 2 for system development [27]. The sensing devices in the bottom layer of the architecture capture and transmit data from or to different IoT services through various network mechanisms, such as Long-Term Evolution (LTE), Worldwide Interoperability for Microwave Access (WiMAX), Wi-Fi, and satellite Internet. Users can access different IoT services by navigating through different Social Network Service (SNS) interface layers. The selected SNS interface should forward the appropriate user’s request to SIoT recommender system to trigger the relevant IoT services. The recommender system not only activates a system call function but also matches the profiles of related SNS users and subsequently recommends possible solutions to the service requestor. 

Several essential perspectives must be considered by the recommender system in SIoT environments. First, the recommender system in a smart service prompts a user to select a social community according to the available choices. Thus, the system establishes a temporary social network where users can share their common interests and relevant information [21]. The recommender system brings together entities with similar objectives. Second, the recommender system collects data regarding service interaction and relationships (“user belief” in this research). Positive interaction records are able to foster trust, community, and an optimistic environment [31]. The system then performs trustworthiness management to yield better direct or indirect recommendations. For instance, even without strong evidence, a user may prefer entity *A* over entity *B* in a domain; however, the recommender engine should utilize indirect observations from other relevant domains to deduce one or more convincing alternatives. This technique is useful for solving the cold start issue [42] because many common attributes are found in different IoT applications and the incomplete information from highly related domains can be compensated for after convergence is achieved through a social network. 

A summary of related works on recommender system with respect to different criteria (e.g., year of publication, data model, recommendation approach, research domain) is shown in Table 1. Most of the selected research papers focus on recommendation approaches that could be adopted in SIoT environment. From the summary, various data model and recommendation approaches are adopted in different research domains. User profiling does not exist in certain personalized recommendation approaches, which may cause difficulty in SIoT implementation. Without a user profile, those personalized recommendation approaches are mainly applied to a specific domain due to the limited data obtained from its user. Besides, the suitability of the approach for trajectory analysis is highlighted in the Table 1 as well. 

#### Use Case Scenario

This subsection describes a use case scenario for the usage of a recommender engine across different disciplines in SIoT applications.

Michael is a university freshman. He is not used to several aspects of the university environment; for example, accommodation access, campus facilities (e.g., library and sports facilities), and transportation. Initially, Michael faces several difficulties simply because he has no idea where to turn for information during difficult situations. By exploiting various social relationships (classmates and other campus communities) that establish through several smart campus applications within the area, Michael finds it easier to adapt himself to the environment. A smart community app that provides personalized recommendations regarding the best place to stay, eat, visit, or play sports is highly valuable under these circumstances. Such an app would be able to provide recommendations to users by considering several factors, such as places near the user’s friends, facilities in the area, and accessibility of transportation. For instance, Michael plans to use the gym at the college at 5:00 p.m. by submitting his booking to the gym center. The booking system is unable to detect that Michael’s classmate John, who lives near his hostel, has also booked a similar time slot. Due to the strong relationship between Michael and John in the social network, sufficient data or beliefs exist to support the proposition that both have similar interests. Thus, the automatic appointment mechanism in SIoT platform will recommend that Michael and John make an appointment to exercise together. The smart community app then launches a carpooling application to solve the transportation problem. By matching users with similar needs, profiles, and perhaps even trajectory patterns, the app can provide freshmen information such as the best bus route and the most preferred restaurants. Such information sharing can be achieved by adopting a reliable intelligent SIoT platform within the campus community. 

### 2.2. Recommendation Methodologies

Recommendation plays an increasingly important role in smart services. Recommendation systems provide users with a list of recommended items (e.g., products or locations) that they may be interested in (preferences) or predict how much a user might prefer each recommended item by assigning a confidence value [43]. Stai et al. [44] classified recommendation systems into four categories, namely content-based recommendation, demographic filtering, CF, and hybrid recommendation, according to the information filtering method used. Each method considers different factors to make a recommendation. For example, a content-based recommendation system [45] considers users’ past records and the characteristics of relevant items during filtering. The demographic filtering system [46] considers user characteristics (e.g., gender and age) as a reference model. The CF system [47] collects and analyzes the preferences and behaviors of users with similar backgrounds and subsequently predicts a list of potential items to recommend. Finally, the hybrid recommendation system [48,49] uses a combination of the aforementioned approaches or integrates other heuristic approaches [50] to overcome the weaknesses of individual methods.

**Figure 3:**
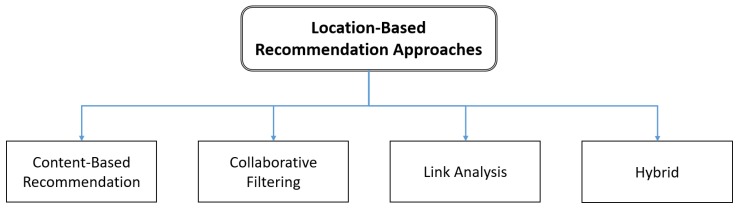
Recommendation methodologies used by location-based recommendation systems.

Studies have indicated that users can exploit information from social networks to improve the accuracy of recommendations [51]. In general, four major recommendation approaches are commonly adopted by social networks for location-based analysis [42], as displayed in Figure 3. Considering the complexity of providing location-based recommendations, location-based recommendation systems (e.g., tourism recommendation systems) mainly provide generalized recommendations [49]. Conventional travel systems regularly recommend places to visit to users according to their travel agent’s opinions and tastes. These agents or experts may occasionally have limited knowledge regarding destinations and places of interest, and the recommendations offered by them may have been suggested by their colleagues within the same travel agency. The aforementioned scenario explains the LA approach [52,53] (an enhanced version of CF) that uses the user–object relationship to identify experienced users (travel agents) and interesting locations. The aforementioned information explains the applicability and benefits of LA in generating a list of recommendations. However, a personalized module is required that can further process the suggestions generated according to user profiles to yield superior recommendations. We believe that a hybrid approach that incorporates personalization is the optimal solution to the recommendation problem because this approach allows the balance between satisfaction and computational complexity to be shifted depending on users’ needs.

#### 2.2.1. Content-Based Recommendation System

The content-based method [22] is the earliest approach to be adopted for use in a recommendation system. The content-based method involves using preferences extracted from user profiles (e.g., age, gender, and favorite activities) and features extracted from location data (e.g., tags and categories) to make recommendations [45]. In the content-based method, accurate and structured information must be extracted from user profiles and location features to obtain high-quality recommendations. Matching information between items (e.g., location) and users is the essential process. Three steps are involved in developing a content-based recommendation system: item representation, profile learning, and recommendation generation [54]. First, user trajectory records are collected through a location-based social network. Stay points (geographic regions where a user has stayed over a certain time interval within a distance threshold) or other features (e.g., the type of location and area) are then used to develop a location’s profile. Moreover, data on users’ preferences and behaviors are collected from the platform to develop user profiles. The required data are explicitly provided by users or implicitly collected. The content-based recommendation system extracts and computes the similarity of the features for the stay points and user to rank the recommendations. A list of the top *N* locations with high similarity are recommended to users.

Content-based recommendation systems are widely adopted when a significant amount of attribute information (feature extraction) is accessible. In general, content-based systems should combine structured (user profiles) and unstructured (text-rich object) attributes when providing recommendations. However, in current IoT environments, performing accurate feature extraction is still difficult due to the high variety in certain domains (e.g., multimedia content and eatery selection). With finite feature extraction and limited domain knowledge, a recommender system can only perform a very shallow analysis. Moreover, content-based systems emphasize matching users to objects that are similar to the objects they have liked in the past and ignore external influences. Such emphasis may lead to overspecialization, where users are restricted to recommendations for only those objects already rated by them [55]. Thus, a group of researchers introduced CF, in which community opinions are aggregated and group intelligence is leveraged to solve the recommendation problem. 

#### 2.2.2. Collaborative Filtering (CF) 

CF overcomes the drawbacks of content-based recommendation systems by integrating community viewpoints. CF is based on the simple idea that if users X and Y rate some common items, their interests are considered similar. During recommendation generation, the recommender engine searches for items that exist in user X’s profile but not in user Y’s profile. These items are then ranked and recommended to user Y [56]. The CF method predicts that a user is more likely to visit a location if their preferences and past histories are similar to those of other users who have visited the location. Two types of CF exist: user-based [47,57] and item-based [58] CF approaches. User-based approaches use similarity measures between each pair of users, whereas item-based approaches use similarity measures between each pair of items [42]. In a location-based social network, the similarities between users or items (e.g., locations or activities) can be inferred from users’ ratings and trajectory histories. 

CF involves three main aspects: user preference, the nearest neighbor, and recommendation generation [56]. Explicit and implicit rating are the two common rating methods used in CF. Explicit ratings are ratings given by a user to items on a sliding scale (e.g., 4/5 stars for a tourist attraction). Implicit ratings suggests user preference indirectly (e.g., page views, clicks, and purchase transactions). After capturing user preferences, the nearest neighbor of a user is located. The Pearson correlation coefficient and cosine similarity are commonly used for distance measurement. The similarity between two users (*user*1 and *user*2) with respect to their preference for an object (*obj*) is usually represented using the similarity metric presented in Equation (1). This similarity metric is used in many recommendation systems for performing trajectory analysis [59,60,61,62]. After generating the list of nearest neighbors, the system recommends the top *N* locations to the user according to the similarity values:

(1) $$UserSim\left(user1,user2\right)=\frac{\sum_{obj\in OBJ}rating\left(obj,user1\right)\times rating\left(obj,user2\right)}{\sqrt{\sum_{obj\in OBJ}rating{\left(obj,user1\right)}^{2}}\;\sqrt{\sum_{obj\in OBJ}rating{\left(obj,user2\right)}^{2}}}$$

People have argued that under the CF model, a new location cannot be recommended to a user until a high number of users have rated the location [55]. Thus, CF suffers from the problem of data sparsity. According to a previous study [63], the CF method poorly handles the cold start problem. The cold start problem occurs when the system must process users or items with limited data. In this scenario, the recommendation model does not have sufficient knowledge to provide recommendations [42]. The user-based CF approach experiences problems in similarity model construction when the number of users is large; thus, the real-time performance of the system is affected. IoT applications always involve large numbers of users and items. The process of similarity model construction in CF can be very time consuming and presents a scalability challenge given the rapid growth of the Internet and wireless communications. Thus, the item-based inference approach is preferred in step 2 of the CF method due to its relatively stable performance and suitability for offline execution. 

#### 2.2.3. LA

LA algorithms, such as PageRank [52] and hypertext-induced topic search (HITS) [53], are widely used enhanced versions of the CF method. These algorithms utilize generic recommendation approaches to rank the large amount of available web content according to the co-citation concept. The concept of co-citation in LA is proposed by Small [64] to measure the similarity between scientific papers. Two papers are co-cited if a third paper has citations to both of them. This suggests the proposition that the author of a scientific paper will cite only papers related to their work. The PageRank value [52] for a page (*u*) is expressed as given in Equation (2). The PageRank value for a page (*u*) is dependent on the ratios between the PageRank values for each page contained in the set *B_u_* (*v*) (*B_u_* refers to the set recording all pages linking to page *u*) and the number of links from page *v* (*L*(*v*)). In this study, we apply LA for location history modeling. We capture the relationship between stay points and model multiple users’ location histories with a tree-based hierarchical graph for further processing. The application of the LA method is detailed in Section 4:

(2) $$PR\left(u\right)=\underset{v\in B_{u}}{\sum }\frac{PR\left(v\right)}{L\left(v\right)}$$

LA algorithms extract high-quality nodes from a complex network by analyzing the network structure. We use network theory to evaluate the connections between network nodes (points of interest in this study), discover anomalies that violate known patterns, and subsequently acquire new patterns from the analysis. The advantages of the LA method are mentioned in the following text. It considers users’ experiences when making recommendations, amplifies ratings from experienced users, and is robust against the cold start problem [24]. However, the LA method has a serious problem when a majority of users has visited most of the considered locations. In this situation, limited information is extracted for further recommendation because no significant pattern can be obtained from the user behavior. To overcome this drawback, a personalized recommender engine can be used to further customize the recommendation according to user preferences. Huang et al. [63] proposed the adoption of a score function where a user who has visited more locations needs to have more overlapping trajectories in order to have greater influential in a recommendation. Although the LA method improves on conventional CF, the issue of the low emphasis on implicit user preferences remains a problem in the LA method. 

#### 2.2.4. Hybrid Approach to Personalized Recommendation

Two or more recommendation methods are commonly combined to improve the accuracy of the recommendation results [65,66]. The combination of content-based and CF methods is one of the most popular hybrid approaches [67,68]. Other famous hybrid models are based on bioinspired and probabilistic methods, such as neural networks [40,69], genetic algorithms [70], deep learning [71], and Bayesian networks [72,73]. Some studies have attempted to combine the aforementioned methods in different forms to obtain superior recommendation results and to overcome the drawbacks of the individual methods. For example, content-based and CF methods require sufficient data regarding past ratings to provide accurate recommendations. We adopt a hybrid method in this study to address problems such as data sparsity and cold starts in CF, low customization for attribute selection in content-based methods, and the lack of personal preference consideration in LA. 

With the advent of location-based social networks, recommendations based on the information (e.g., check-ins and location trajectory information) collected through these networks have attracted considerable research attention. Due to user preferences being highly accessible through social network interactions, personalization mechanism used by the recommender engine is of particular research interest. As mentioned in a previous study [74], most personalization systems are based on some type of user profile. These user profiles may contain static and dynamic data, which may align with short- or long-term user preferences. User profiles are generally represented as sets of weighted keywords [75], association rules [76], semantic networks [77], or weighted concepts [23,78]. The framework used in this study uses the dynamic form of user profiles because these profiles can grow with the implicit and explicit information collected from users and reflect changes in their preferences and behaviors. Furthermore, we adopt a tree-based hierarchical model with weighted concepts to represent user preferences (as described in Section 4.3). A hierarchical model is selected rather than a flat set model because the hierarchical model enables the collection of a large volume of data that are more generic [78]. When using the hierarchical model in user profiling, users can select the hierarchy level to obtain different amounts of detail; this enables a flexible recommendation. For instance, users can select a location from higher levels, such as “country,” or lower levels, such as “town,” according to their needs.

Zheng et al. proposed a hybrid recommendation system that integrates the location correlation method of LA models into the Slope One algorithm to achieve an effective and accurate item-based CF model [24,25] that can provide generic and personalized recommendations. In their system, a popularity score and hub score are assigned to each location and user, respectively, to track each user’s travel expertise. A ranking of expert users and interesting locations is computed through a mutually reinforcing relationship. Zheng et al. extended the HITS algorithm to identify experienced users and interesting locations in their hybrid recommendation model. The framework proposed in this paper integrates the location history model of Zheng et al. [24] with our user profile and knowledge construction module to deliver personalized recommendations in the smart campus application UniCAT [23].

UniCAT is used for several campus services, such as information sharing and dissemination, e-commerce, location navigation, and social networking [79]. Because of the positive initial finding, an in-depth investigation is conducted on the application of the proposed framework to a smart community. Compared with user check-in data, also known as point location check-in data, used by social network services (e.g., Facebook and Foursquare), user-generated trajectories contain richer information and can be used to more accurately estimate user preferences. The experimental results reveal the possible application of the proposed user trajectory framework in several SIoT domains. An experiment is conducted [39] to explore the feasibility of personalized recommendation adoption in SIoT domain. The paper presents a generic SIoT architecture that is suitable for a smart campus. The successful implementation of the model leads to a more comprehensive study of personalized recommendation and its performances in this paper.

The framework proposed in this paper is based on domain-independent user trajectory analysis, which can be adopted for any IoT application. We integrate personalized recommendation into the location-based social network by assisting each user to understand their surrounding (points of interest; POIs) with the knowledge collected from community records. Moreover, we maintain a sufficient level of uniqueness by filtering the recommendations according to the user profiles (e.g., user preferences and histories). The correlation between user trajectory analysis, personalized recommendation and SIoT environment is illustrated in Section 3 to provide the reader an overall view of our system implementation. Possible challenges of system implementation is discussed in the next section as well.

## 3. User Trajectory Analysis

**Figure 4:**
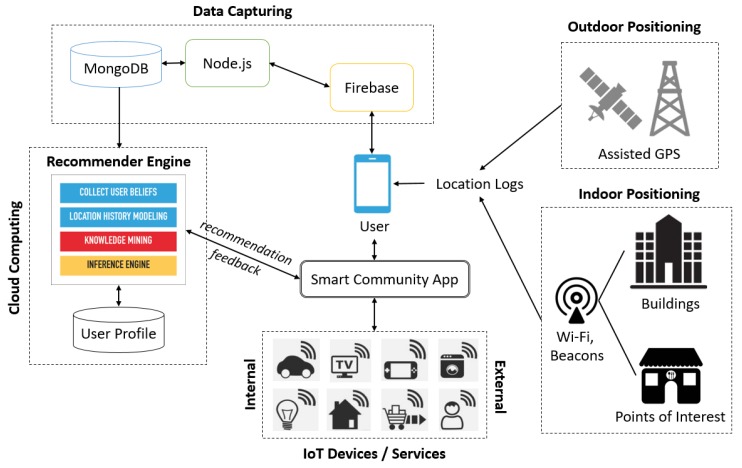
Overall SIoT architecture.

**Figure 5:**
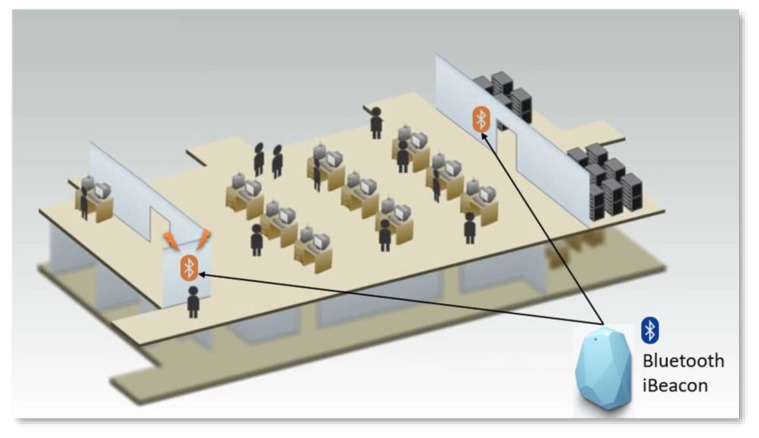
Indoor positioning with Bluetooth iBeacons.

Figure 4 illustrates the overall implementation of personalized user trajectory analysis within a smart community. User movement trajectories are collected through indoor and outdoor positioning data. A common Global Positioning System (GPS)–enabled device is used to obtain location information from a satellite network. An assisted GPS is used for obtaining location information when the network device is in a location where the penetration of satellite signals is limited. Information for indoor positioning is obtained from Bluetooth beacons installed on the walls or ceilings of buildings and POIs [80]. Several Estimote iBeacons (available from: https://developer.estimote.com/ibeacon/) are deployed at the main entrance of buildings (Figure 5) to capture the indoor trajectories of users. We adopt Wi-Fi-based trajectory alignment and calibration [81] to improve the accuracy of indoor positioning. Data from the location logs are delivered to Firebase (mobile and web platform) for further processing.

**Figure 6:**
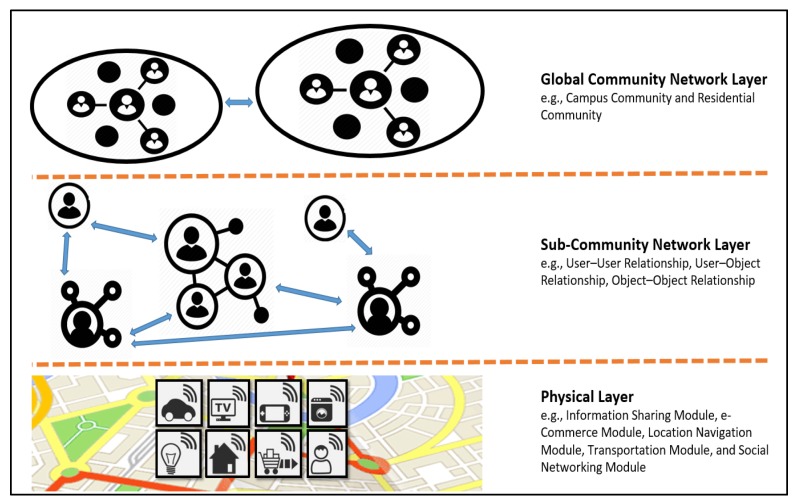
SIoT community model.

Figure 4 also indicates that the internal and external IoT devices and services can communicate with each other through web service calls (e.g., REST and SOAP) as well as the UniCAT smart community app. At the physical layer, the fundamental IoT modules (e.g., the information sharing, e-commerce, location navigation, transportation, and social networking modules) embedded with sensing, actuating, processing, and networking capabilities can offer different types of services that can be used by users and things to accomplish everyday activities, as displayed in Figure 6. Modern societies are heterogeneous, dynamic, and complex. People engage in interactions and establish unique social relationships with each other in communities developed according to several factors (e.g., common objectives, interests, needs, and influence). Social networking users interact and collaborate with each other to solve complex problems. Applications providing interactive and collaborative features are called SNSs. The concept of social networking can also be applied to IoT ecosystems. The social features of the IoT paradigm have given rise to a new concept of social networking with smart things and services, which is referred to as SIoT. The current high worldwide penetration of IoT applications has significantly increased the interaction between users and things. Thus, relationships are established not only between users but also between smart things and services. 

An abstraction layer called the subcommunity network layer, which exists between the physical and global community layers, allows users and things from different communities to establish user–user, user–object, and object–object relationships based on several factors [9,21], such as common interests, common goals, friendships, and common owners. Service discovery plays an important role in SIoT environments because users and things require an efficient recommendation system to reduce the system load.

The recommender engine generates recommendations for users through the integration of various inputs from internal and external SIoT services. For instance, a dining place is recommended on the basis of trajectory analysis that takes into consideration a restaurant ranking provided by a social networking service (e.g., Foursquare). User personal preferences should be considered for the filtering of recommendations. As per the framework displayed in Figure 4, we adopt NoSQL to capture most of the system data, such as the location logs, user transactions, object interactions, and user preferences. The data are stored on cloud computing platforms (e.g., Google Cloud Platform and Amazon Web Services) for further reference. Section 4 presents the details of the recommender engine.

### Issues and Challenges

The application of personalized recommendation in a smart community has considerable potential for future SIoT applications. Intelligent service discovery can produce new solutions to meet the growing and varying requirements of users. However, the following challenges remain to be addressed:

(1) Data collection: An efficient data-capturing model is required to represent different levels of diversity in user beliefs and the social relationships of users.
(2) Inference engine: A generic yet tailored approach (from generic to personalized) is required to offer users various customizable outcomes for recommendation.
(3) Dynamicity and scalability: A recommendation system that can be applied to different problem domains is required. The system should scale out or scale in when no direct evidence supports an outcome.

The following section describes how the aforementioned challenges are addressed with the proposed personalized recommendation framework based on the KDI model. 

## 4. Personalized Recommendation for Smart Communities

**Figure 7:**
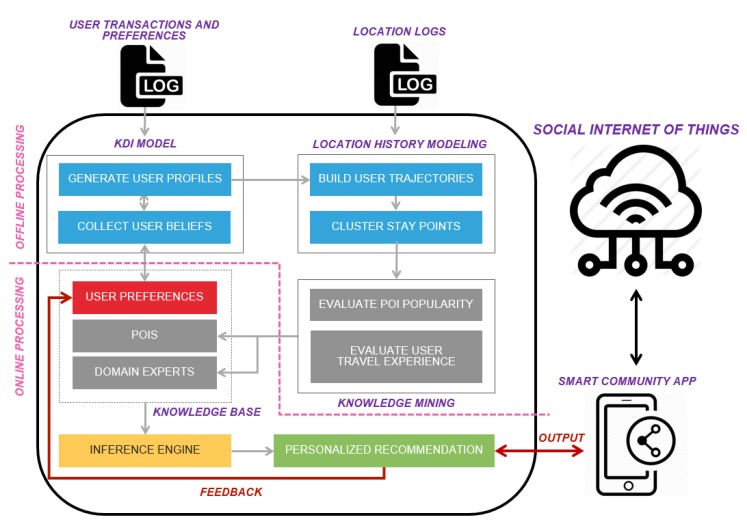
Proposed personalized recommendation framework (an extension of our work in [23]).

Figure 7 displays the overall architecture of the proposed personalized recommendation framework, which is an extension of our research work in user trajectory analysis [23]. We adopt the knowledge–desire–intention (KDI) model [23] to collect user data explicitly (e.g., ratings for items) and implicitly (e.g., location history and number of orders) to profile users. The collected user profiles contained data that are filtered and sorted using the KDI model according to the assigned weight and confidence level. The KDI model yields a hierarchical representation of user data that allows different IoT applications to be integrated with it. In contrast to many conventional recommendation systems, the proposed model allows different types of commonly available smart community user data, such as data related to food preferences, daily activities, purchasing records, and other factors, to be stored inside a user profile. We propose a novel hybrid approach that involves filtering location-based recommendations tailored to users’ preferences. We apply link analysis (LA) [24,52,53], which is an enhanced version of collaborative filtering (CF) [26], on user trajectories to generate the top *N* recommendations for various smart community services (e.g., information sharing and transportation). A filtering process is then performed according to the user profiles obtained using the KDI model. This approach combines the advantages of LA, which allows offline preprocessing of time-consuming and costly tasks prior to reasoning, and user profiling through the KDI model, which allows the generation of real-time personalized recommendations, to support SIoT environments that involve numerous dynamic user–object social relationships. The uniqueness of the proposed framework lies in the fact that it combines filtering and reasoning mechanisms to generate personalized recommendations in an SIoT environment.

Our proposed framework comprises the KDI modeling module [27], location history modeling module, knowledge mining module, knowledge base, inference engine, and most importantly, the personalized recommendation module. As mentioned in earlier, we adopt the LA method proposed by Zheng et al. [24] for the location history modeling and knowledge mining modules. The KDI modeling, location history modeling, and knowledge mining modules are executed offline in the proposed framework and preprocess user location logs and preferences. These offline components require higher load than the reasoning module, which is operated online. The personalized framework does not require the offline modules to be executed for every recommendation. The collected logs and preferences (both old and new) are reprocessed only after a certain amount of time (*T_p_*). Let *T_p_* be a dynamic threshold value that is determined by the percentage of new data added into the user profile and trajectory. For instance, when the accumulated new data (logs or preferences) are more than 2% of the total records, the weightage of the user beliefs and POIs must be updated to better reflect the user’s knowledge. Different metrics can be used to determine *T_p_*, which is based on environment factors. If updating is performed too frequently, the updates will not reflect the changes in human behavior because such changes usually require time to emerge. 

### 4.1. Data Capture

The proposed system captures user or object data, such as location history, transaction, and preference data, which are converted into user or object profiles for use in KDI modeling and location logs for use in location history modeling. As displayed in Algorithm 1, the user data are first converted into location points (LPs) and location logs. For each object, the user or object profiles include the time spent at the LPs; the point frequency, which is the number of visits made to an LP; the point recentness, which is the most recent time the user visited the LP; and the LP velocity, which is displacement per time. The aforementioned data serve as the input of the KDI and location history modeling modules for further analysis.
**Algorithm 1.** DataCapturingInput: User transactions and preferences, userPrefOutput: Generated user profiles and location logs1LP = locationPointDetection(userPref);2userLogs = locationLogsGeneration(userPref);3**Foreach** user **do**4  TS = durationCalculation(user, LP);5  PF = pointFrequency(user, LP);6  PR = pointRecency(user, LP);7  LV = locationPointVelocity(user, LP);8  userProfiles.add(TS, PF, PR, LV);9**Return** userProfiles, userLogs;

### 4.2. KDI Model

Bloedorn et al. [78] suggested the use of a hierarchical model rather than a flat set model for user profile because a hierarchical model enables the recommendation system to be more generic in capturing a variety of data. The hierarchy levels can be fixed or dynamic according to user preferences. A simple user profile can be constructed from a reference taxonomy, and a complex profile can be constructed through a reference ontology. The aforementioned statement is in line with the proposed user profiling approach. In this research, user profiles are the user or object information recorded in the smart campus application. The profiles include the user or object preferences, location histories, and personal information provided by the users. The user or object preferences are fed into a belief system, which is based on the belief–desire–intention (BDI) model. Other profiling methods, such as weighted keywords, semantic networks, weighted concepts, or association rules, can also be used in the proposed framework [74]. We select the BDI model because this model is a type of computational model that resembles human reasoning [82]. The KDI model, which is an advancement of the BDI model, advocates and emphasizes that human beliefs are the fundamental elements on which human decisions are made [23]. In our experimental design, user preferences are set as beliefs that constitute a tuple with three attributes (item, weights, and level) and their corresponding values. Item refers to the smallest unit of data (e.g., color, place, or age) in a user or object profile, and weight represents the importance of the data unit through the calculation of parameters such as frequency, recency, and fixity. We adopt hierarchical belief modeling [27] to represent progressive levels of belief. This strategy is different from that used in conventional content-based methods. Three levels, namely temporary belief (raw data), analyzed belief (information), and permanent belief (knowledge), are assigned to each data unit by referring to its confidence vector that accumulates over time. The personalized recommendation framework utilizes and compares the relevancy of objects according to the given beliefs when making any decision. Feedback from every action taken is collected explicitly from the user to update the weight of each belief in every recommendation attempt.

**Figure 8:**
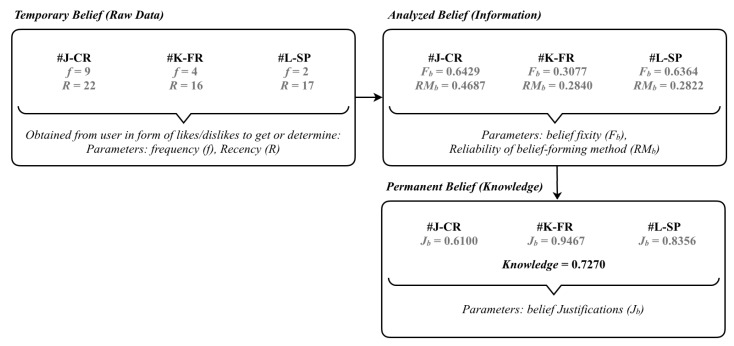
Application of the KDI model for assigning weightage to each user’s preferences.

In Figure 8, *#J-CR*, *#K-FR*, and *#L-SP* represent a user’s beliefs in the *#J*, *#K*, and *#L* domains, respectively. At the level of temporary belief, raw data are obtained from user records collected through interaction with various smart community apps. The frequency (*f*) and recency (*R*) of each belief are captured to determine their relative importance index. Only beliefs that achieve a certain level of importance (beyond a threshold value) are selected for further analysis. At the level of analyzed belief, a weight is assigned to each propositional belief, which is represented by the belief fixity (*F_b_*). Let *F_b_* indicate the confidence level of each user’s belief on the proposition. Moreover, an indicator of the reliability of a belief-forming process, *RM_b_*, is calculated. This parameter indicates the degree to which a set of beliefs is formed from a reliable or truth-conducive belief-forming process. The parameters *F_b_* and *RM_b_* are used for escalating the relevant beliefs to the next level. At the level of permanent belief, the output from the previous stage is used to obtain the Gettier-centered justification (*J_b_*) for each belief. Belief justification is the process of validating that a belief is connected to truthfulness and not to luck or coincidence. The threshold level of knowledge (*K*) is calculated to determine the ‘preferred’ and ‘nonpreferred’ beliefs. For instance, any belief with *J_b_* greater than or equal to *K* is selected and ranked in the knowledge base. A similar action is also performed for the LPs in the user trajectories. Then, the knowledge threshold (*K*) becomes the reference point for all belief justifications in our knowledge base. 

All beliefs are dispositional because they may be based on assumptions, fallacies, or impulses (all characterized by chance or uncertainty) and hence surrounded by doubts [27]. A model or system should not be completely reliant on beliefs surrounded by doubts. Therefore, any decision or output produced by beliefs cannot be completely relied on [83]. The KDI model aims to address this important drawback of a typical BDI model by considering knowledge as a more suitable element of reliable human decision-making than belief. The refinement or processing stages that the belief system undergoes in the KDI model are summarized and embedded in the proposed personalized recommendation framework. 

### 4.3. Location History Modeling

In our personalized user trajectory recommender system, location history modeling involves deriving user trajectories from user location histories, as displayed in Algorithm 2. Location logs obtained from user location histories contain collections of GPS points. These points are connected sequentially according to their time series, and the GPS data are split into trajectories if the time interval between consecutive points exceeds a certain threshold (*∆T*). A tree-based hierarchical graph (*TBHG*) is used for modeling multiple users’ location histories [24]. A TBHG integrates two structures, namely a tree-based hierarchy and a graph, on each level. The tree-based hierarchy (*H*) is a collection of stay-point-based clusters (*C*). The tree indicates the parent–children relations at different levels, and the graph indicates the peer relations among nodes at the same level.
**Algorithm 2.** LocationHistoryModelingInput: Collection of users GPS logs, userLogsOutput: Tree-Based Hierarchical Graph (TBHG)1**Foreach** user **do**2  trajectory = LogParsing(userLogs);3  S = StayPointDetection(trajectory);4  LocH = PersonalLocHis(S); *//individual user*5  SP.add(S);   //collection of stay points6H = HierarchicalClustering(SP);7**Foreach** level **do** //build a graph on each level8  **Foreach** user **do**9    g = graphBuilding(g, LocH);10    G.add(g);11TBHG = (H, G);12**Return** TBHG;

The trajectories are then converted into stay points. Stay points are geographic regions where a user has stayed over a certain time interval within a distance threshold. The dataset includes the stay points detected from users’ trajectories. By using a density-based clustering algorithm, the dataset is hierarchically clustered into some geospatial regions. Similar stay points from various users are assigned to the same clusters at different levels. Directed edges connect the tree-based hierarchy with users’ trajectories and clusters at the same level. If consecutive stay points on one path are individually contained in two clusters, a link is created between the two clusters in a chronological direction according to the time series of the two stay points. These clusters represent POIs. 

### 4.4. Knowledge Modeling

For knowledge modeling, a HITS-based inference model is used to infer users’ travel experiences (hub score) and location interests (authority score) in a region, as depicted in Algorithm 3 (adapted from [24]). HITS is a search-query-dependent ranking algorithm that is often used for web information retrieval. In the knowledge model, a user’s visit to a POI (cluster) is considered a directed link from the user to the location.
**Algorithm 3.** LocationHistoryInferenceInput: TBHG=(*H,G*) and users’ location histories, *LocH*Output: Users’ hub scores, **S** and locations’ authority scores, **A**.1**S** = **A** = ∅;2**For***i* = 1; *i < |L|; i ++ //on each level*3  **For**
*j* = 1; *j < |C|; j ++ //on each cluster on the level*4    **For**
*k* = *i* + 1; *k ≦ |L|; k ++ //on each sub-level*5      C = LocationCollecting(*k, c, H*)*;*6      M = MatrixBuilding(*C, LocH*)*;*7      (*x,y*) = HITS-inference(M)*;*8      **S** = (x)*;*9      **A** = (y)*;*10**Return (S,A)**;

A user is a hub if they have visited many locations, and a location is an authority if it is frequently accessed by many users. By using a power iteration method, final scores are generated for each user and location. A user has multiple hub scores for different regions. Moreover, a location has multiple authority scores specified by its ascendant clusters at different levels because each cluster of the TBHG specifies an implied region for its descendant clusters. The calculations for the hub and authority scores are performed offline to ensure the efficiency of the recommendation system:

(3) $$S_{ij}^{l}=\underset{u_{k}\in U}{\sum }A_{lq}^{k}\times v_{ij^{’}}^{k\;.}$$

(4) $$A_{lq}^{k}=\underset{c_{ij}\in c_{lq}}{\sum }v_{ij}^{k}\times S_{ij^{’}}^{l\;.}$$

Adjacent matrices (*M*) are constructed between users and locations according to the user access to the locations, which belong to the same ascendant cluster. A mutual reinforcement relationship exists between user travel experience (*A*) and location interest (*S*) in Equations (3) and (4). The subscripts *i* and *j* denote the *i*th level parameter of the *j*th cluster in the TBHG, *S^l^* represents the *l*th location interest, and *A^k^* represents the *k*th user travel experience. The score for each location sequence within a given region is calculated according to the travel experiences of users traversing the sequence and the locations of interest in the sequence. Because multiple paths begin from a location, the location interest is shared among all these paths. The location interest in different paths is influenced by the probability of users taking these paths. The results from knowledge modeling are location sequences that contain high scores. The resulting domain knowledge consists of interesting POIs and opinions of domain experts.

### 4.5. Recommendation Module

An inference engine is used in the recommendation module to make recommendations. The KDI model used in our personalized recommendation framework records the dataset in binary form to represent users’ likes and dislikes with respect to various propositional beliefs. This strategy allows the system to determine the frequency or number of belief occurrences. The time of visit is captured to determine the recency of users’ last visit to the object of belief. By using the theory of degrees of beliefs, belief fixities (*Bf*) (firmness or tenacity of beliefs) and vulnerabilities to doubts (*VtD*) are used in reaching the next stage of analyzed beliefs. The resulting knowledge value recorded in the knowledge base as well as the authority score is used by the inference engine to generate the recommendation, as displayed in Algorithm 4. Because the proposed framework aims to provide a generic reference model for implementation, users can also consider other bioinspired [69,70,71] and probabilistic [72,73] methods to replace the inference engine. We must balance the complexity and applicability of the selected model in generating real-time recommendations based on different domain requirements.
**Algorithm 4.** KnowledgeInferenceInput: User-selected *region* and knowledge baseOutput: Sorted collection of POIs.1**For***i* = 1; *i < |L|; i ++ //on each level*2  **For**
*j* = 1; *j < |C|; j ++ //on each cluster on the level*3    **If**
*region*.contain(C[j])4      A.add(C[j].authority);5      SP.add(C[j].poi);6**Foreach** SP **do**7  k = KnowledgeKDI(SP);8  K.add(k);9POI = SIoT-inference(SP, A, K);10**Return***POI*;

According to the pseudocode (Algorithm 4), when a geospatial region is specified by a user, the inference engine determines the corresponding level of hierarchy in the TBHG and then retrieves the POIs (clusters) in the specified region. The authority scores of the clusters and the corresponding knowledge values obtained from the KDI model are retrieved from the knowledge base and used for ranking the POIs [23]. Users can submit their satisfaction with each recommendation as feedback to the inference engine and knowledge base. The weight vectors for the relevant beliefs can be further fine-tuned according to users’ requests. This function ensures that the problem of personalized recommendation within a fixed boundary does not occur in the proposed framework. 

The complexity of the recommendation algorithm is analyzed to investigate the possibility of its real-time implementation. Because the KDI modeling module is the core module of the proposed framework, the following analysis is performed. According to the pseudocode in Algorithm 4, POIs are searched for travel data according to their ascendant clusters (*j*) at different levels (*i*). Subsequently, the inference engine filters irrelevant POIs by referring to the beliefs and weights in the KDI knowledge base. Assume that *C* and *L* are the maximum number of clusters and levels, respectively. According to the aforementioned explanation, the complexity of (*i*, *j*) is *O*(*m*) and that of POI filtering is *O*(*n*). Therefore, the complexity of the overall knowledge inference algorithm for a personalized recommendation based on the KDI approach is *O*(*m + n*). The aforementioned analysis indicates that complexity does not become a negative factor that affects the real-time implementation of the recommendation algorithm in SIoT environment. The highest complexity of the proposed framework occurs during location history modeling. The complexity of the algorithm is *O*(*x*^2^*y*^2^) during the construction of a TBHG with *x* levels for *y* users. However, TBHG construction is a data preprocessing stage that is only revisited by the framework after sufficient new input is obtained in the knowledge base by referring to a threshold value (*T_p_*). The time taken for every recommendation requested by a user is determined through the online operation of the inference engine only. Thus, the complexity of real-time recommendation is linear (*O*(*m + n*)) in the proposed framework. 

## 5. Implementation and Measurement

To evaluate the effectiveness of the proposed recommendation framework, the GeoLife [28], Weeplaces [29], Brightkite [30], and Gowalla [29] public datasets (as shown in Table 2) are used to determine the precision and recall ratios. The GeoLife dataset includes tracking data for 182 users in Beijing over 3 years. The dataset comprises 17,621 recorded trajectories. Each trajectory log is a sequence of time-stamped points that contains latitude, longitude, and altitude information.

Weeplaces is a website that aims to visualize users’ check-in activities in Location-Based Social Networks (LBSNs). The Weeplaces dataset is generated using data crawled from Foursquare. The dataset contains 971,309 POIs generated by 15,799 users. Brightkite is an LBSN that enabled users to check-in to places and see who else had visited the location. Brightkite is acquired and its operations discontinued by Limbo. The Brightkite dataset is collected through Brightkite public Application Programming Interfaces (API) and consists of data from 4,491,143 check-ins by 58,228 users. Gowalla is an LBSN that was acquired by Facebook in December 2012 [30]. The Gowalla dataset includes user profiles, location profiles, and check-in history collected prior to 1 June 2011, through the Gowalla public APIs. The dataset contains 2,844,076 POIs generated by 319,063 users:

(5) $$p=\frac{tp}{tp+fp}$$

(6) $$r=\frac{tp}{tp+fn}$$

To calculate the precision and recall ratios, datasets must be divided into training and testing sets. In this study, the data for the final 8 months are included in the testing set and the remaining data are used for training. The training set is used to learn user preferences and construct the recommendation model. The system is then evaluated by examining whether it could suggest sites visited by a user within the querying region according to the training data:

(7) $$F=\frac{\left(\beta^{2}+1\right)\;\cdot \;S\left(precision\right)\times S\left(recall\right)}{\beta^{2}\;\cdot \;\left(S\left(precision\right)+S\left(recall\right)\right)}$$

Precision, given by Equation (5), is the fraction of all recommended items that are relevant, and recall, given by Equation (6), is the fraction of all relevant items that are recommended. Precision (*p*) and recall (*r*) are measured as proportions of true positives (*tp*), false positives (*fp*), and false negatives (*fn*). The parameter *F*1 is the weighted average of precision and recall. Both false positives and false negatives are considered in calculating *F*1 [Equation (7)] [84]. The positive real *β* entails the selection of *β* such that recall is *β* times as important as precision. The parameter *F_β_* determines the effectiveness of retrieval with respect to a user who attaches *β* times as much importance to recall as precision. 

### Baselines and Methods

Ranking by frequency (RF) method: The more frequently people access a location, the more interesting this location might be. The visiting frequency of a location is the ratio between the number of users visiting the location and the time span of their visits (i.e., from the first day at least one user accesses the location to the last day at least one user accesses the location).

LA: LA is a generic recommendation approach in which a location is more likely to be recommended if a higher number of experienced users (expert users) have visited the location. 

Hybrid approach: This approach integrates LA with a KDI model to provide personalized recommendations based on user preferences.

## 6. Experimental Results

To examine the user satisfaction of the proposed trajectory analysis method, we use four public datasets, namely the GeoLife, Weeplaces, Gowalla, and Brightkite datasets, for benchmarking. Precision–recall analysis is performed to measure and compare the performance of the proposed recommendation method, LA method, and RF method. The RF method is used as the baseline method (i.e., it acted as a reference for the other two approaches). LA is a conventional approach in which most of the time recommendations are applied. The proposed hybrid approach integrates the advantages of LA and also employs a user belief system, namely the KDI model, to support the personalization process. Thus, the hybrid approach should outperform the other approaches.

### 6.1. Comparison of Precision and Recall Measurements in Individual Datasets 

**Figure 9:**
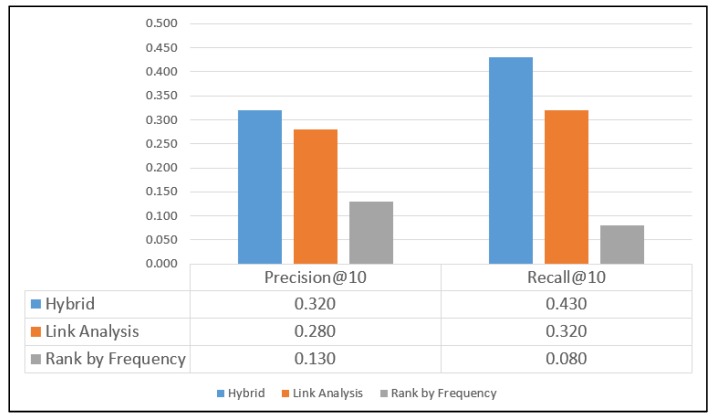
Results for the GeoLife dataset.

Figure 9 displays the results obtained using the GeoLife dataset. The performance of the hybrid and LA methods for the GeoLife dataset (Figure 9) is inferior to that for the other datasets. 

This result is obtained because the GeoLife dataset does not include a ready list of POIs (unlike the other three datasets) and POIs are generated from user stay points obtained from user trajectory logs. The GeoLife dataset mainly includes location data for Beijing over 3 years. The dataset comprises 17,621 trajectories generated by 182 users. The proposed hybrid approach outperformed the LA and RF methods when POIs are generated from user location logs. 

**Figure 10:**
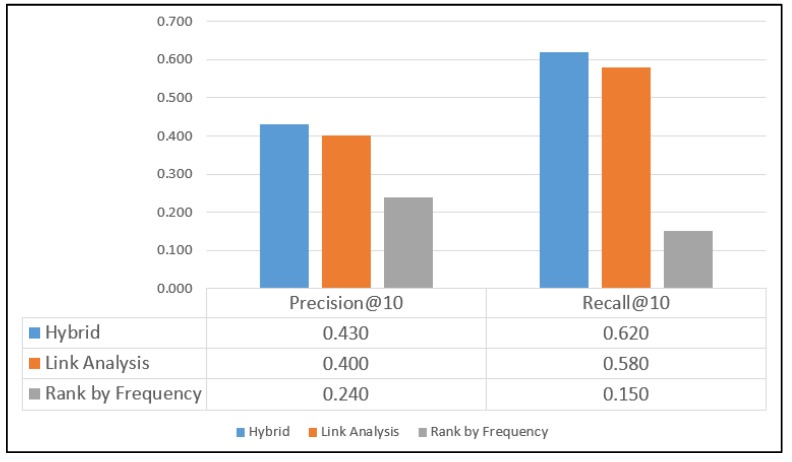
Results for the Weeplaces dataset.

The Weeplaces dataset comprised data crawled from the Foursquare platform, which is a location data platform famous for its city guide application. The dataset contains 971,309 POIs for 7,658,368 check-ins generated by 15,499 users, with most check-ins being concentrated in a specific region. The Weeplaces dataset is larger than the GeoLife dataset. However, because the Weeplaces dataset includes suitable POI data, higher precision and recall values are obtained for the three methods with the Weeplaces dataset than with the GeoLife dataset. As depicted in Figure 10, the precision values of the hybrid, LA, and RF methods are 0.43, 0.40, and 0.24, respectively, and the recall values of these methods are 0.62, 0.58, and 0.15, respectively. Thus, the hybrid method outperformed the other two methods.

**Figure 11:**
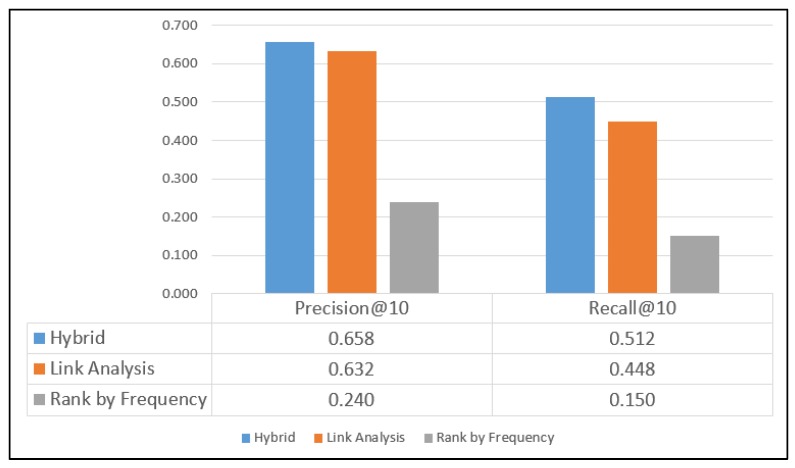
Results for the Gowalla dataset.

**Figure 12:**
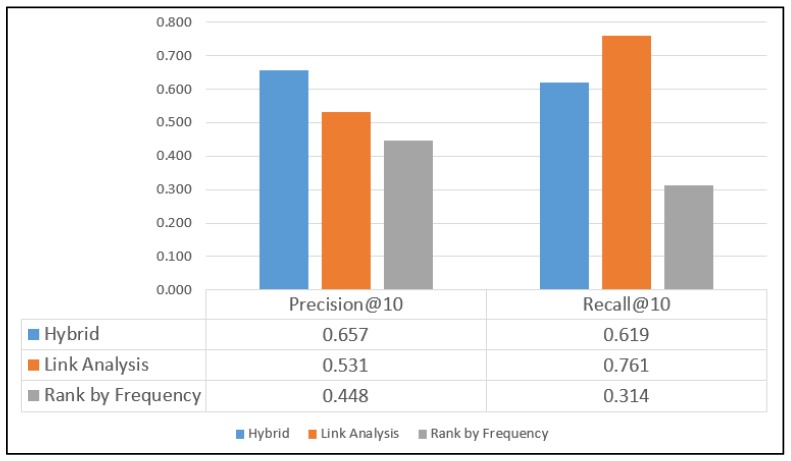
Results for the Brightkite dataset.

Brightkite and Gowalla are large LBSNs. The collected user and location profiles in the Brightkite and Gowalla datasets comprise well-maintained and appropriate descriptive information. These datasets are collected through public APIs for locations throughout the world. The Brightkite dataset consists of 4,491,143 check-ins generated by 58,228 users, and the Gowalla dataset contains 36,001,959 check-ins generated by 319,063 users. Thus, the Gowalla dataset is the largest of the four datasets used. Similar to the Weeplaces dataset, the Brightkite and Gowalla datasets also provide POIs, which helped to increase the precision and recall rates for the three adopted methods. The proposed hybrid approach marginally outperformed the conventional LA method for the aforementioned two datasets, as displayed in Figure 11 and Figure 12.

For the Gowalla dataset, precision values of 0.658, 0.632, and 0.240 are obtained with the hybrid, LA, and RF methods, respectively, and for the Brightkite dataset, precision values of 0.657, 0.531, and 0.448 are obtained with the three methods, respectively. For the Gowalla dataset, recall values of 0.512, 0.448, and 0.150 are obtained with the hybrid, LA, and RF methods, respectively, and for the Brightkite dataset, recall values of 0.619, 0.761, and 0.314 are obtained with the three methods, respectively. 

### 6.2. Measuring F1 for GeoLife, Gowalla, Weeplaces, and Brightkite Datasets 

**Figure 13:**
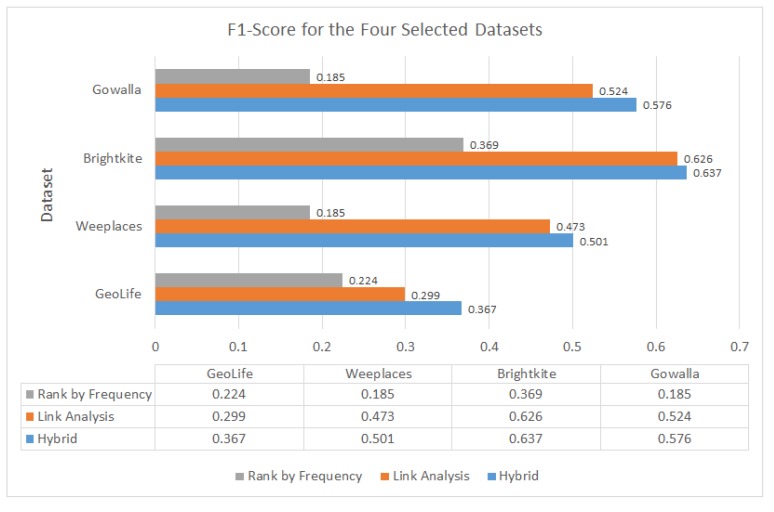
*F*1 values obtained with the three methods.

The results in Figure 13 accord with the proposed hypothesis that a user belief system can benefit the recommendation. As displayed in Figure 13, the average *F*1 values of the proposed hybrid approach are up to 27.95%, 3.98% higher than those of the RF and LA methods for the four adopted datasets. An improvement of 3.98% may not seem impressive, but we should analyze the overall performances of the methods from various perspectives to determine the significance of the improvement. 

First, the proposed method outperformed the other two approaches over four datasets, where each dataset represented different domains in the actual environment. The proposed approach is adaptable to different data conditions. Second, a “personalized” recommendation method that captures user or object preferences over time should gradually improve the recommendation accuracy of the system. Such improvement can be achieved because the inference engine can utilize more information over time to make a precise recommendation. One possible issue that may arise over time is the over-tuning of the inference engine with overloaded information. However, a feedback mechanism and satisfactory monitoring from users can easily help prevent this problem. 

Significantly lower precision and recall scores are obtained for the RF method than for the other two approaches. This result is obtained because unlike the other two methods, the RF method disregards individual user experiences. The proposed hybrid approach marginally outperformed the LA method due to the integration of the KDI model, which considers not only experienced users’ trajectories but also user behaviors simultaneously.

## 7. Conclusions

In this article, a personalized recommendation framework suitable for SIoT is proposed. A use case scenario in a smart community is used to describe SIoT deployment. The overall architecture of user trajectory analysis, which includes the indoor and outdoor positioning modules, internal and external IoT devices and services, the data-capturing module, the recommender engine, and the multilayer SIoT community model, is described in detail. For further analysis, the proposed personalized recommendation framework for smart communities is decomposed into several modules, namely the KDI modeling module, location history modeling module, knowledge mining module, knowledge base, inference engine, and personalized recommendation module. The proposed hybrid recommendation algorithm is implemented in a smart community with several SIoT applications and services. User trajectories over several years are collected through different community services, such as e-commerce and location navigation services. The collected data and the selected benchmarking datasets are used for performance analysis. Three recommendation approaches, namely the RF method (baseline approach), LA method (conventional approach), and hybrid method (proposed approach), are examined in this study. According to the precision and recall results, the proposed personalized recommendation method achieved an average of up to 28% higher satisfaction for users compared with the other two approaches. Thus, the proposed method provides more accurate user recommendations than the other approaches do. 

The proposed personalized recommendation algorithm is based on user profile and belief systems that might not exist in some recommender engines with simple designs. In a future study, we plan to focus on trustworthiness management to ensure reliable interactions between users and thing to reduce exposure to malicious objects. Several addition topics, such as the extension of smart community coverage by providing recommendations for locations with different cultures, time-slots and environmental settings, can be examined in future studies. Deep learning can be used to model SIoT behaviors for delivering suitable recommendations in service discovery and composition. 

## Figures and Tables

**Table 1:** Summary of research publications on recommendation approaches in SIoT.

Research Publication	Year	User Profile	Data Model	For Trajectory Analysis	Type and Recommendation Approach	Dataset	Domain
Zheng et al. [24,25]	2009, 2011	No	Tree-based hierarchical graph	No	Item-based collaborative filtering: (Personalized)	GeoLife [28]	Travel Advisory
Ning et al. [13]	2017	No	Temporal and spatial segments	Yes	Inner-line anomaly detection method: (Generic)	Data collected through mobile crowdsensing	Smart Vehicle
Luan et al. [36]	2017	No	Tensor partition	No	Collaborative tensor factorization: (Personalized)	Data collected from Weibo and DianPing	Location Based Social Network
Ning et al. [14]	2017	No	Node-centric generation tree	Yes	Trajectory-based interaction time prediction algorithm: (Generic)	Simulation	Smart Vehicle
Luan et al. [37]	2018	No	Two-level POI category hierarchy structure	No	Maximal-marginal-relevance method: (Personalized)	Data collected from Weibo	Location Based Social Network
Amin et al. [38]	2018	Yes	Social network structure	No	Statistical, Louvain and Greedy methods: (Generic)	Egonets-Facebook	Smart Community
Lye et al., [23,39]	2017,2019	Yes	Tree-based hierarchical graph and KDI Model	Yes	Trajectory-based KDI-link analysis: (Personalized)	UniCAT [23] and Weeplaces [29]	Smart Campus
Huang et al. [40]	2020	No	Multi-attention network	No	Multi-attention based neural network: (Personalized)	Data collected from Meetup, and MovieLens-1M	Social Networks
Chen et al. [41]	2020	No	Time aware SIoT knowledge graph	No	Item-based collaborative filtering: (Personalized)	MIT	Smart Community
Proposed Framework	2020	Yes	Tree-based hierarchical graph and KDI Model	Yes	Trajectory-based KDI-link analysis: (Personalized)	GeoLife, Weeplaces, Gowalla [29], and Brightkite [30]	Smart Community

**Table 2:** Comparison of different datasets with respect to number of users, records, POIs and time span of the collection.

Dataset	Type of Record	Number of Users	Number of Trajectories / Check-in Points	Number of POIs	Time Span of the Collection
GeoLife	GPS trajectory	182	17,621	-	36 months
Weeplaces	Check-in point	15,799	7,658,368	971,309	92 months
Brightkite	Check-in point	58,228	4,491,143	772,764	31 months
Gowalla	Check-in point	319,063	36,001,959	2,844,076	29 months
